# Gene co-expression network based on part mutual information for gene-to-gene relationship and gene-cancer correlation analysis

**DOI:** 10.1186/s12859-022-04732-9

**Published:** 2022-05-24

**Authors:** Yi-Hua Jiang, Jie Long, Zhi-Bin Zhao, Liang Li, Zhe-Xiong Lian, Zhi Liang, Jia-Rui Wu

**Affiliations:** 1 Hefei National Laboratory for Physical Sciences at Microscale and School of Life Science, University of Science and Technology of China, Hefei, China; 2 Chronic Disease Laboratory, School of Medicine, South China University of Technology, Guangzhou, China; 3 Key Laboratory of Systems Health Science of Zhejiang Province, Hangzhou Institute for Advanced Study, University of Chinese Academy of Sciences, Chinese Academy of Sciences, Hangzhou, 310024 China

**Keywords:** Correlation analysis, Direct association, Part mutual information, Multiple omics integration, Cancer survival analysis

## Abstract

**Background:**

Finding correlation patterns is an important goal of analyzing biological data. Currently available methods for correlation analysis mainly use non-direct associations, such as the Pearson correlation coefficient, and focus on the interpretation of networks at the level of modules. For biological objects such as genes, their collective function depends on pairwise gene-to-gene interactions. However, a large amount of redundant results from module level methods often necessitate further detailed analysis of gene interactions. New approaches of measuring direct associations among variables, such as the part mutual information (PMI), may help us better interpret the correlation pattern of biological data at the level of variable pairs.

**Results:**

We use PMI to calculate gene co-expression networks of cancer mRNA transcriptome data. Our results show that the PMI-based networks with fewer edges could represent the correlation pattern and are robust across biological conditions. The PMI-based networks recall significantly more important parts of omics defined gene-pair relationships than the Pearson Correlation Coefficient (PCC)-based networks. Based on the scores derived from PMI-recalled copy number variation or DNA methylation gene-pairs, the patients with cancer can be divided into groups with significant differences on disease specific survival.

**Conclusions:**

PMI, measuring direct associations between variables, extracts more important biological relationships at the level of gene pairs than conventional indirect association measures do. It can be used to refine module level results from other correlation methods. Particularly, PMI is beneficial to analysis of biological data of the complicated systems, for example, cancer transcriptome data.

**Supplementary Information:**

The online version contains supplementary material available at 10.1186/s12859-022-04732-9.

## Introduction

In the era of big data, life science research has produced vast amounts of data from different aspects of the biological systems. These aspects are usually referred to as omics such as genomics and transcriptomics. The complexity of a biological issue, such as cancer progression, requires integration and systematic analysis of data from multiple omics. Current studies mainly center on gene expression because of their direct contributions to the phenotype of the research object. A study often aims to find which gene can be used as a target to perturb a biological system of interest. In this case, the correlation pattern of gene expression will be the key for the multi-omics integration as it is either the results of the regulations from other omics or the reasons contributing to the states of other omics. The correlation pattern of gene expression, also referred to as a correlation network, is usually represented as an indirect graph where the genes are nodes and the inter-gene correlations are edges. The edges in a correlation network are defined by correlation measures, such as the Pearson Correlation Coefficient (PCC), and Spearman Correlation Coefficient (SCC), etc. To obtain important genes from the correlation network, a frequently used method is to extract network modules. A network module on the gene correlation network is a set of genes that have high internal correlation but are relatively isolated from the other parts of the whole network. A set of genes forming one network module indicates that they may be regulated by similar mechanisms and contribute to common biological functions. This module level correlation analysis has been remarkably successful in interpreting gene expression data. The most popular algorithm for network module extraction is the Weight correlation network analysis (WGCNA). WGCNA uses correlation measures such as PCC to initialize the whole network, and uses the topological overlay measures for module extraction. This method can give robust results at the module level [1].

However, the module level interpretation of gene expression data lacks detailed analysis at the gene-pair level. The implementation of biological function relies on the cooperation of genes. The basic functional unit of a biological system is the relationship between a pair of genes defined by omics. For example, a transcriptional regulation relationship is a transcription factor regulates a target gene. The protein–protein interactome consists of thousands of relationships of two proteins that interact with each other. Obviously, the expression pattern of genes is highly related to those omics defined relationships. Hence, analyzing the correlation pattern of gene expression at the gene-pair level is necessary to better understand biological functions and to enable multiple omics integration. In the human genome, the real expression relationships among genes contains cascading structures, and interweaves into a network. When using correlation measures like PCC, there will be many dense clique-like structures in the resulting correlation network, while the real active biological relationship may not be that densely organized. This redundancy of edges (gene-pairs) prevents us from finding the real important gene or inter-gene relationships. In the meantime, PCC-like correlation measures, such as the SCC or biweight midcorrelation are restricted for linear or monotonic correlations. But the real biological relationship may not be satisfied by this mathematical assumption.

Partial or semi-partial(part) statistics may be helpful to eliminate redundancy. To detect nonmonotonic association, Mutual Information (MI) is a theoretical solution. To overcome these two restrictions at one time, we should use a MI-like multivariate statistic for correlation analysis. Part Mutual Information (PMI) is a recently invented algorithm that satisfies our requirement. PMI has shown better performance on simulated data based on yeast gene regulatory network than many other algorithms including direct association like Conditional Mutual Information (CMI) [2]. PMI calculates the correlation network in a very unique way. PMI starts with a fully connected network and proceed with checking every PMI value among all combinations of variables then retains only the top one [2]. Compared to methods like the partial/part correlation matrix calculation implemented by R package ppcor, the PMI network calculation procedure may intrinsically consider the heterogeneity of distribution of correlation strength. The resulting networks are also sparser [3]. All these mathematical features are combined. The theoretical advantage of PMI for biological correlation analysis is promising. But as a new algorithm, PMI has not been widely used in real data. The high calculation complexity makes it hard to apply PMI to data with the scale of the human genome. In the meantime, switching from PCC to PMI in the analysis of correlation patterns of biological data raises questions in the multi-omics viewpoint, e.g., what is direct biological association, and how a direct biological association is different from an indirect association, when connecting them to biological mechanisms stemming from multiple omics. The mathematical concept of direct association has indicated that the variable-pairs found by PMI are more important than those found by PCC, because the later one is indirect and redundant. We need to understand this difference in a biological context.

In this paper, we apply PMI network calculation procedure to analyze the correlation of mRNA transcriptome data of human cancer and confirm that PMI direct associations are more biologically significant than PCC indirect associations. We also apply the PMI-based correlations for cancer survival analysis.

First, we construct PMI networks for the differential co-expression status of chosen gene sets using a multiple-stage breast cancer dataset. The results suggest PMI is more efficient in capturing important gene-relationships. We calculate PMI networks from several subtype cancer datasets, and compare the recall of gene correlation networks using PMI or PCC to the omics defined gene-pair relationships. We find that PMI is more efficient in recalling these relationships, and that the recalled relationships in the PMI network have significantly higher omics defined weights than those in the PCC network. Finally, we extracted gene-pairs in PMI networks that match with gene-pair relationships defined by copy number variation or DNA methylation in cancer. We then test their individual influence on patient survival, and build a simple scoring system to classify patient survival. Our results suggest PMI can extract more biologically important results at the gene-pair level than PCC. The PMI network calculation procedure can provide networks sparse enough and are biological meaningful for further detailed analysis at the level of variable pairs. PMI-based correlation analysis will be more helpful in the study of complex biological systems with multiple omics.

## Results

### PMI correlation change network for cancer progression

In the first part, we want to test the performance of PMI extracting biological meaningful gene-pairs in an intuitive way. We want to obtain an optimal PMI network for each input data, the PMI network calculation procedure will be repeated with gradually increased thresholds until the resulting networks are stable. We collect a breast cancer mRNA transcriptome dataset, which contains 2 cancer progression with 4 stages. If we directly use the whole genome data to calculate an optimal PMI network, the calculation will be too much to finish. To obtain the optimal PMI networks, we divide the whole genome into small gene sets by WGCNA module extraction and GO enrichment analysis provided by WebGestalt [1, 4]. Then, we calculate the optimal PMI networks of one gene set on each stage of one cancer progression and combine the four networks into one integrated network, which hereafter is referred to as the PMI correlation change network.

**Fig. 1:**
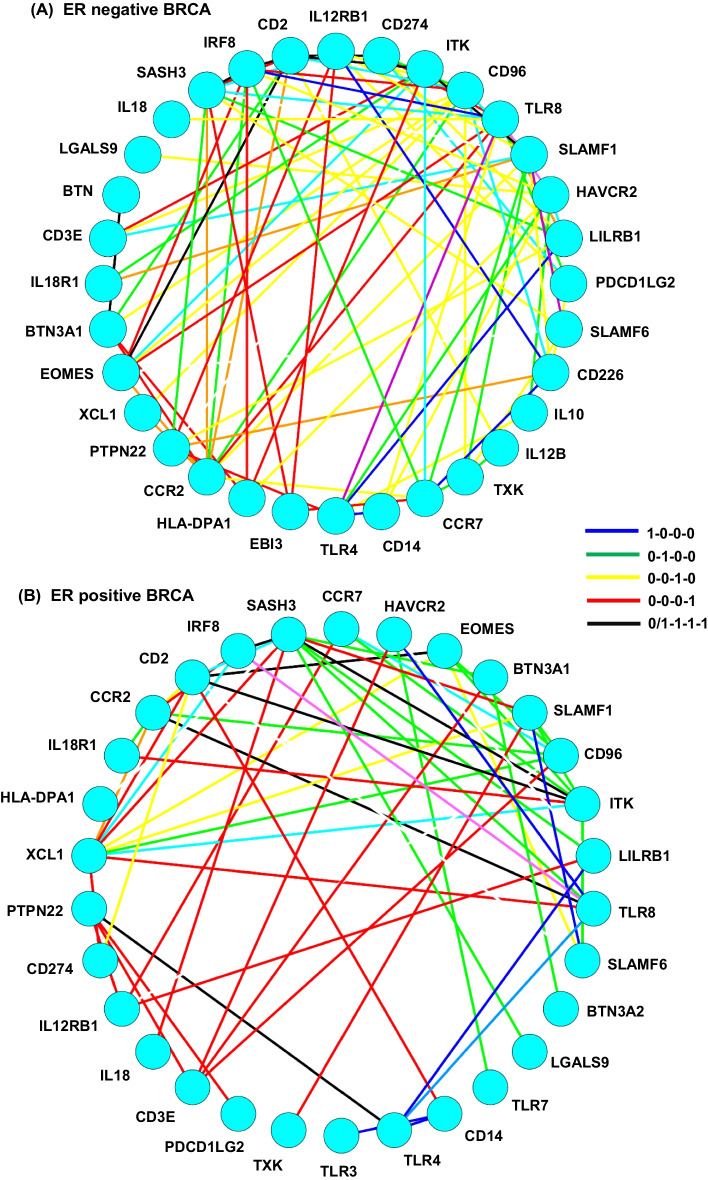
The PMI correlation change network of GO: 0032609. The PMI correlation change network for GO: 0032609 production of interferon-gamma in ER negative process (**a**) and ER positive process (**b**). The nodes are placed by the mean stage values of the edges of them from 6 o’clock position by count clock direction. The colors of edges represent the different development stages. Edges in one stage only are colored with main colors blue (normal), green (cancer stage 1), yellow (cancer stage 2) and red (cancer stage 3). Edges in all 4 stages or 3 cancer stages are colored with black. Edges with inconsecutive correlations along the 4 stages are colored by white and are ignored

To find the important parts involved in cancer development, we exam a variety of detailed structures in a correlation change network and review literatures that report their functions in cancer development. In this part, we use the correlation change networks of GO:0032609 production of interferon-gamma as an example. This GO term is enriched in all stages of ER negative and positive processes (Fig. 1) [5]. When we place the nodes by the mean stage values (the average of stage numbers of one node’s edges), the well-known immuno-suppressive genes can be observed in the late phase, such as EBI3 in ER negative network, and CD274 (PD-L1) and PDCD1LG2 (PD-L2) in ER positive network [6–8]. There will be several hub nodes in one correlation change network, in which some nodes have edges that exist in different stages. These hub node genes, in combination with their edge stage patterns, reflect the changes during cancer development. For example, XCL1, a chemokine ligand for T cell attraction [7], has 9 neighbors in the ER positive network. XCL1 forms a connection with tumor suppressors: IRF8 and ITK in early cancer stages [9, 10]. In later stages, CD274 (PD-L1), an immunosuppressor, correlates with XCL1. The correlation change network of GO term interferon-gamma production gives a reasonable representation of the biological mechanisms of cancer progression.

**Fig. 2:**
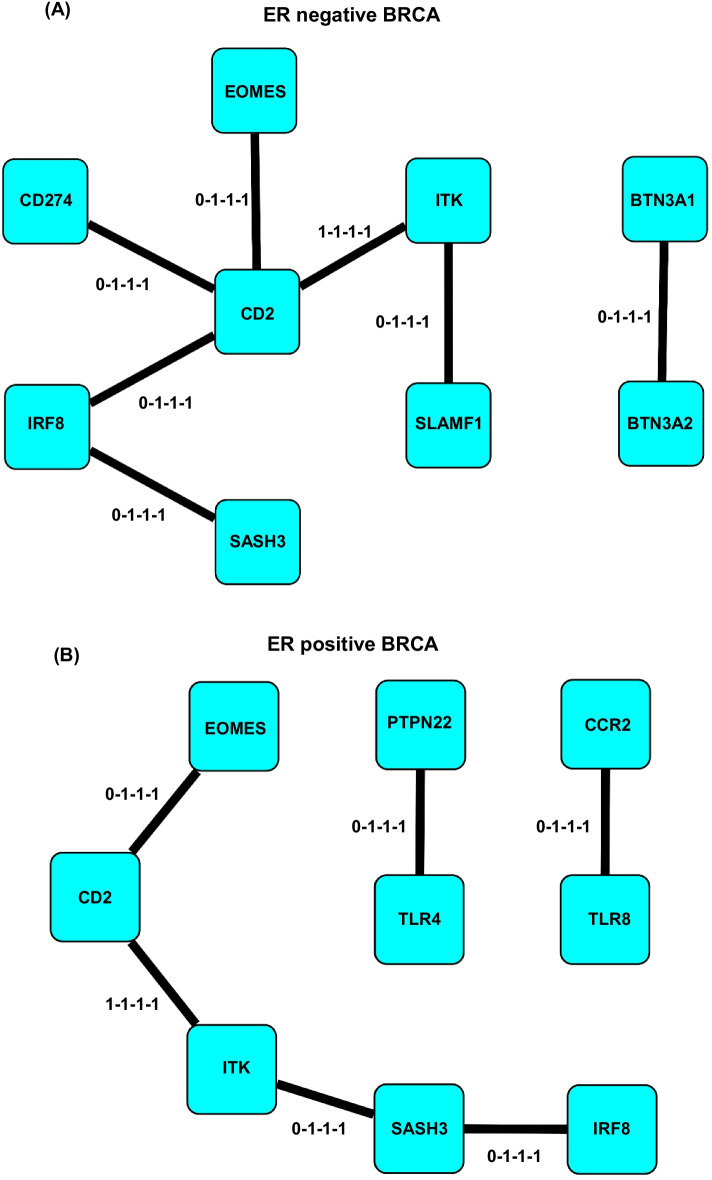
The essential modules of GO: 0032609. The essential modules of PMI correlation change network for GO: 0032609 production of interferon-gamma in ER negative process (**a**) and ER positive process (**b**). We use 4 digits of 0/1 connected by dash to represent which stages does one edge exist. For example, 0–1-1–1 means this edge exists in all 3 cancer stages but not in normal

In the meantime, we address the performance and reliability of an optimal PMI network. Specifically, we would like to confirm that the optimal PMI network possess the essential part of the correlation in the PCC network for the same input data, even if the optimal PMI network is much sparser than the PCC network. Similar to the expression of housekeeping genes in a genome, a set of genes, if they are functionally involved in broad and a variety of different biologic conditions, will have some constitutive correlations. In the two PMI correlation change networks of GO term interferon-gamma production, we observe similar network modules that represent the core function of this GO term. These modules consist of 5 genes (CD2, ITK, SASH3, EOMES and IRF8) with similar stage composition and topological structure (Fig. 2). CD2 is an antigen expressed in all peripheral T cells [7]. ITK is an essential kinase for T cell differentiation and proliferation [7]. So, a constitutive correlation between CD2 and ITK is reasonable and represents the existence of T cells. SASH3 is an adaptor protein, probably downstream of the TCR [11]. EOMES is an essential transcriptional factor (TF) for the differentiation of effective CD8+ T cell [7]. IRF8 is an interferon regulatory factor with tumor suppressive function [9]. As these 3 genes all relate to (or affect) T cell activity in disease situation, it’s reasonable that they connect with the CD2-ITK center in cancer stages but not in normal situations. Similar network modules can be found in other GO term networks. Their existence suggests optimal PMI networks can capture biological mechanisms and are effective in giving reproducible results.

### PMI correlation is closer to omics defined relationship

We match edges from PMI networks and comparable PCC networks with multiple types of omics defined gene-pair relationship, and compare the recalls between PMI and PCC. The results of the comparison are summarized in Table 1, the details are listed in supplementary tables. In normal-status omics, including TR, CoTR, PPI and SCGD, both PMI and PCC networks nonrandomly recall these relationships in almost all cancer subtypes. PMI-recalls have higher nonrandomness and higher weights than comparable PCC-recalls, suggesting PMI networks captured more important parts than PCC networks. TR and CoTR are directly associated with gene expression. But there are too few PMI-recalls of TR in the whole network (Additional file 2: Table S1). Although the PMI-recalls of CoTR cover a fair share of each network, the unrecalled parts are significantly larger than the recalled parts, suggesting the CoTR relationships may lack specificity (Additional file 3: Table S2, Additional file 4: Table S3). On the other hand, PMI-recalls of SCGD have more edges and significantly lower distance values than the PCC-recalls (Additional file 5: Table S4). This contrast suggests that the proximity of a pair of genes is one of the differences between PMI direct associations and PCC indirect associations. For PPI, the PMI-recalls have more edges and higher nonrandomness than the PCC-recalls in all cancer subtypes (Additional file 6: Table S5).

**Table 1:** Summary of PMI-PCC recall comparison

GenePairRelationship	NonrandomlyRecall	HigherNonrandomness	HigherWeights
TR	Yes	Yes	Not defined
TFCoTR	Yes	Yes	Yes
miRCoTR	Yes	Yes	No
PPI	Yes	Yes	Not defined
SCGD	Yes	Yes	Yes
CoCNV-GG	Yes	Yes	Yes
CoCNV-LL	Yes	Yes	Yes
CoCNV-GL	No	May exclude	No
COCNV-LG	No	May exclude	No
CoDM-HH	Yes	Yes	Yes
CODM-LL	No	Yes	Yes
CoDM-HL	No	No	No
CoDM-LH	No	No	No

Similar contrast between PMI and PCC is also observed in disease-specific omics. We look the 4 types of CoCNV (GG, LL, LG and GL). For one pair of genes in one sample, PMI-recalls of GG and LL types have higher nonrandomness and higher weight than comparable PCC-recalls in almost all cancer subtypes (Additional file 7: Table S6, Additional file 8: Table S7, Additional file 9: Table S8, Additional file 10: Table S9). While the GL and LG types seem to be nonrandomly excluded by PMI in some cancer subtypes, this tendency of exclusion is less significant in PCC-recalls (Additional file 7: Table S6, Additional file 8: Table S7). For the 4 types of the CoDM, only the HH type is nonrandomly recalled by PMI networks from each sample in most cancer subtypes, while the nonrandomness of recall of the other 3 CoDM types is not very consistent across cancer subtypes. PMI-recalls of the HH type of CoDM have higher nonrandomness and higher weight than comparable PCC-recalls in most cancer subtypes (Additional file 11: Table S10). The LL, HL and LH types of CoDM also have higher weights in PMI-recalls than PCC-recalls (Additional file 12: Table S11, Additional file 13: Table S12, Additional file 14: Table S13, Additional file 15: Table S14).

In summary, PMI networks recall relationships with higher nonrandomness and higher weights than comparable PCC networks, in both normal-status omics and cancer-specific omics. These observations prove that the PMI network is generally closer to omics relationships, although it is not determinable for one specific PMI edge that is dominated by one specific omics defined relationship. We demonstrate that the mathematical concept of direct association is more biologically significant.

### Cancer survival analysis based on PMI network

**Fig. 3:**
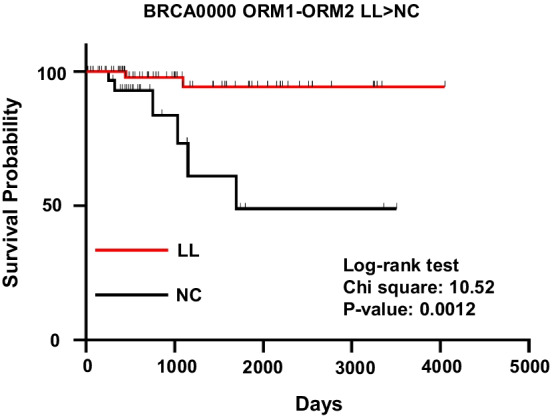
An example of significant CoCNV survival factor. In triple negative breast cancer, patients with ORM1 and ORM2 double loss mutation (LL group, 60 samples, blue) have better survival than those with ORM1 and ORM2 not changing together group (NC group, 35 samples, orange)

**Fig. 4:**
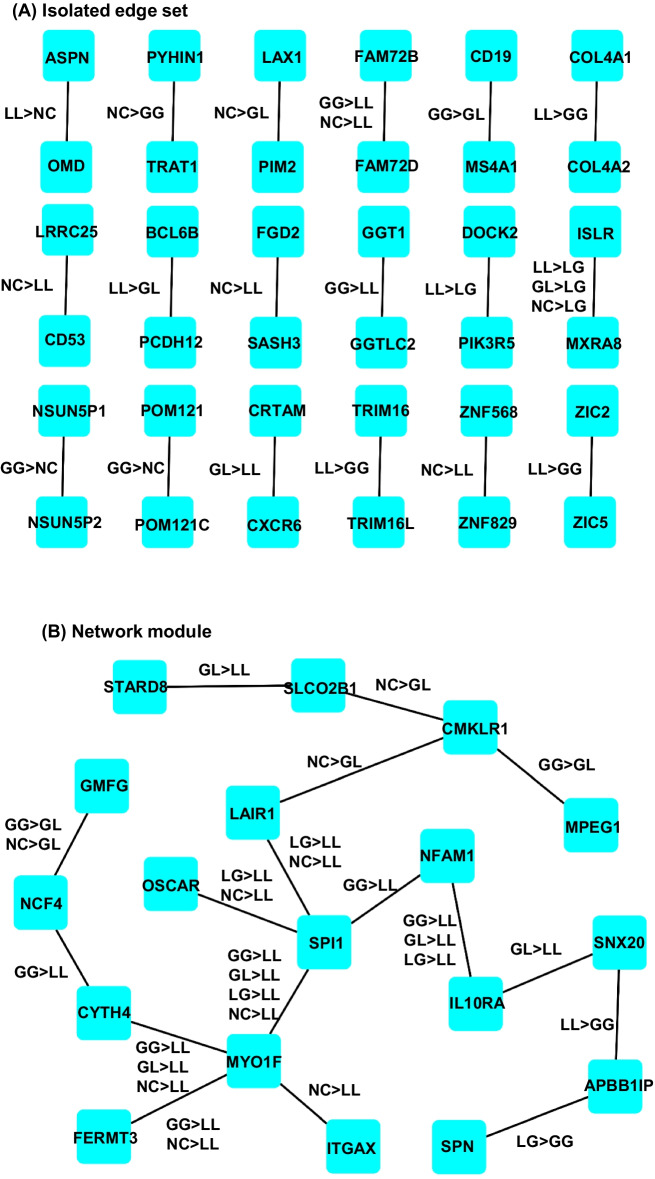
Two types of organization of sets of significant CoCNV survival factors in LUSC00. Survival factors can be a set of isolated edges (**a**) or form into small network module (**b**). The combinations of CoCNV mutation types that make significant survival factor are listed as the label of edges

**Fig. 5:**
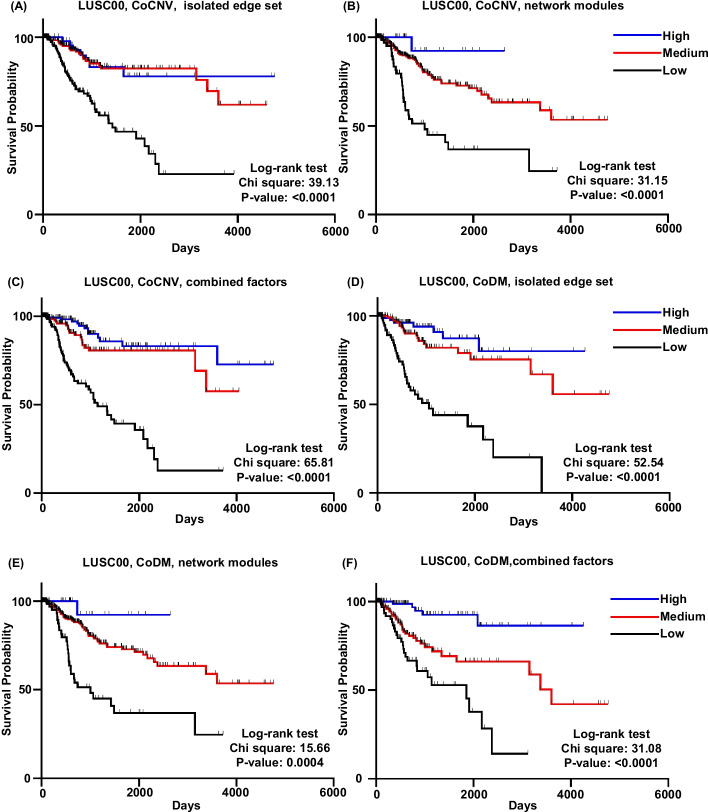
Using more significant factors gives better classification of survival status. The survival status comparison using significant CoCNV/CoDM survival factors organize into isolated edges (**a**/**d**), small network module (**b**/**e**) or combined both of two sets (**c**/**f**) in lung squamous cell carcinoma. For both CoCNV and CoDM, using more factors (**c** and **f**) gives better classification of patient survival

We perform univariate survival analysis with the gene-pairs of CoCNV or CoDM from PMI networks. Patients are divided into groups by the CoCNV or CoDM types of one gene-pair, two groups of patients with significant different survival give one significant survival factor. Such as a CoCNV survival factor: Gene1-Gene2 GG > LL, a CoDM survival factor: Gene3-Gene4 HH-high > HH-low. Many significant CoCNV or CoDM survival factors exist in most cancer subtypes (Additional file 16: Table S15, Additional file 17: Table S16, Fig. 3). The CNV and DM status of one gene directly influences the gene transcription. So, the CoCNV and CoDM survival factors should impact patient survival through the changes of gene expression. However, the gene expression levels between groups divided by these factors are not significantly different. When we use the median value of the expression of these genes to divide patients into low and high expression groups, the survival statuses of the two groups are usually not significantly different from each other. The CNV and DM provide unique information about patient survival that cannot be superseded by expression level. The gene-pairs of significant CoCNV or CoDM survival factors can be a set of isolated edges scattered on the network or some small modules on the network (Fig. 4). Both isolated edges and small modules of factor sets can be used to calculate the scores for patients and divide the patients into groups with significant differences of survival. The score calculated with more survival factors gives better divided patient groups with survival probability (Fig. 5). Cox multivariate analysis with the scores and clinical covariates shows both the CoCNV scores and CoDM scores are stronger classification variables than clinical covariates, and the two of them can be used together to achieve better classification (Additional file 18: Table S17, Additional file 19: Table S18). Only the LUSC00 has sufficient samples for subsampling remodel test, the distribution of *p*-values of the covariates confirmed the consistency of results (Additional file 20: Table S19, Additional file 21: Table S20). So, the PMI-recalled gene-pairs with CoCNV or CoDM relationships are systematically related to the probability of cancer survival, and they may influence the mechanisms for cancer progression.

In the meantime, we find a notable difference between CoCNV and CoDM. For CoCNV, some genes in the PMI network gene set can classify patients into groups with significantly different survival probability by their single gene CNV, such as CTLA4, ORM1, and RASAL3 in triple negative breast cancer. When switching from single gene CNV to gene-pair CoCNV, many genes have improved significance of survival classification (Additional file 22: Table S21). For example, in triple negative breast cancer, PTPN7 cannot separate patients into groups with significant survival difference. The *p*-value of survival test between PTPN7 gain group and PTPN7 unchanged group is 0.25. When cooperating with RASAL3, the PTPN7 and RASAL3 GG group has a significantly better survival probability than the NC group with p-value 0.02. This GG group is also more significant than the division by single gene RASAL3 (*p*-value = 0.03). This result suggests that the overall influence of CNV in one cancer subtype is an accumulation of the single CNV of each gene.

In term of CoDM, the genes that can classify patient survival by their single gene DM never show up in the significant CoDM survival factors in all cancer subtypes. For example, there are 13 single-significant genes and 47 pair-significant genes in triple negative breast cancer without overlap. This result suggests that the impact of DM in one cancer subtype consists of two independent parts, a single-gene part and a pair-of-gene part.

## Discussion

Correlation analysis plays an important role in the analysis of biological data. Most frequently used algorithms focus on obtaining module level interpretation from only one type of data. In case of multiple omics integration, there is a need to find important pairs of variables (edges) from the correlation networks. Current methods in frequent use for correlation analysis give too dense correlation networks. The edges in a dense correlation network contain too much redundancy to highlight important pairs of variables. The direct association and causal interaction are two ways to deal with redundancy. Among direct associations, PMI has a very unique calculation procedure that checks all combinations of the input variables, which may be helpful to get more meaningful results for biological data. Causal interaction usually refers to the directed relationship from TF to target gene. This kind of algorithms often rely on special restriction for the structure of co-expression network or take prior knowledge of whether a gene is TF. The results of causal interaction detection are supposed to be restricted in the field of transcriptional regulation [12]. Considering the above-mentioned two points, the PMI network calculation procedure seems to be an ideal method to detect generally important parts of the biological system. Due to the high calculation complexity and unique calculation procedure, PMI has not been widely used in the analysis of biological data. The performance of PMI networks on real experiment data needs to be tested. Also, we are interested in learning the differences between PCC indirect associations and PMI direct associations as well as their connections to biological relationships. In this paper, we first trialed PMI on expression data of WGCNA-GO enriched small gene sets. We show that optimal PMI networks can display the whole correlation pattern with fewer edges while retaining important core biological relationships. This will be very helpful to uncover important correlations of a pair of genes. So, using PMI to refine the results from traditional algorithms is promising.

Based on the assumption that the correlation pattern of gene expression data partly matches the relationships defined by omics, we match edge lists from PMI/PCC networks and compare their recall status. Although a single specific edge in the PMI network may not fully and precisely match one omics defined gene-pair relationship, the whole PMI network is statistically closer to many types of omics defined gene-pair relationships, as compared to the comparable PCC network. So, PMI direct association is indeed more biologically significant than PCC indirect association. A PMI-based correlation network can better represent the correlation pattern of the biological data and is more helpful for multi-omics integration.

**Fig. 6:**
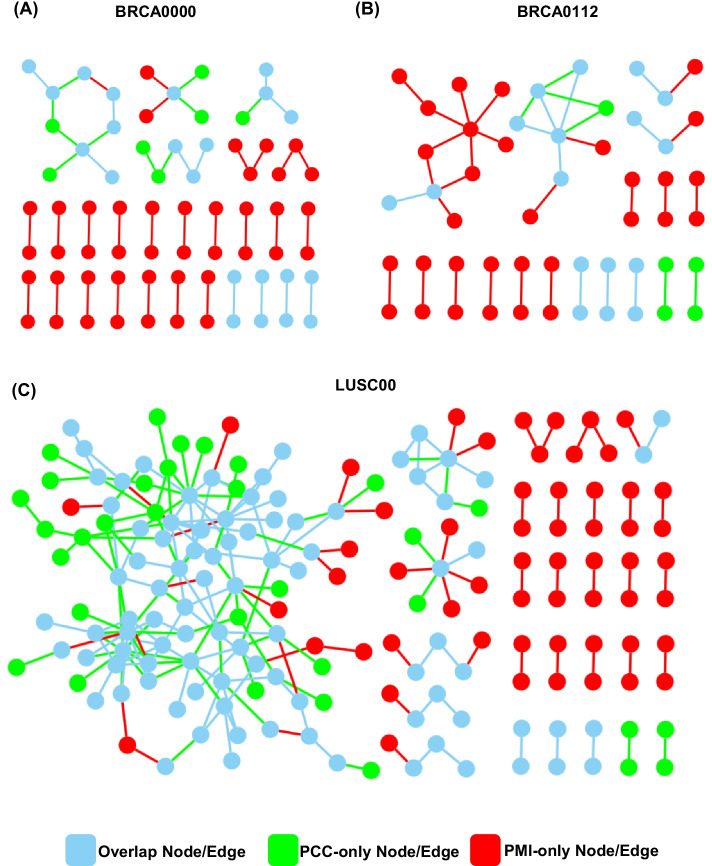
The PCC-only survival factors are dispensable on the network viewpoint. The networks of PMI and PCC significant CoCNV survival factors combined for triple negative breast cancer (**a**), triple positive breast cancer (**b**) and lung squamous cell carcinoma (**c**). Red nodes and edges are PMI-only, green nodes and edges are PCC-only, the rest node and edges are from both PMI and PCC. It is shown that, irrelevant to their scales or structures, PMI covers most parts of the combined networks. Removing PCC-only parts do not change the biological meaning of the whole networks. These figures focus on the structure of networks, gene names of nodes are not listed. Similar results are observed in CoDM networks, they are too large and are shown in supplementary materials

In the last part, we use gene-pairs with CoCNV or CoDM relationships from the PMI/PCC networks of cancer subtypes to classify patient survival. In the case of the log rank test statistic values of single survival factors, PMI does not show significantly better classification than PCC. When we put the gene-pairs of both PMI and PCC significant survival factors on a network, we see that PMI covers more isolated edges than PCC. Excluding the PCC-only (Green in Fig. 6, Additional file 1: Fig. S1) edges, the network does not lose too much module structure. So, when selecting the same number of edges, using PMI-based network can cover more groups of genes with different functions, which will represent the mechanisms of the entire biological system better.

The ideal multiple omics integration should be to fully model the correspondence from the biological relationships to the mathematical relationships of the biological variables in one biological condition. Although we have proved that PMI-based gene correlations are significantly closer to biological relationships than PCC-based results, we still cannot precisely map gene correlations and biological relationships. This ideal modeling may be approached by using data with less heterogeneity and higher specificity in a biological condition. On the other hand, finding the more important parts in the biological system is the major task of correlation analysis. For complex human diseases like cancer, the changes from normal status to disease status are often driven by the combination of a vast number of possible mutations scattered on the whole genome, instead of one single dominating factor. In this situation, PMI will be a better choice for the correlation analysis.

Our current study focuses on the performance and reliability of a whole calculation procedure for correlation analysis of real biological data. We do not include more comparison with other types of statistics for direct association, because the advantage of PMI network calculation procedure may be the result of combining PMI core algorithm definition and the cutting edges by comparing combination numbers of PMI calculation and the threshold scanning strategy. The design and modification of these calculation procedure involve too much mathematical and computational work, which is beyond our expertise. On the other hand, we guess the edge-cutting method is the key for refining edge-level correlation results, but it also causes the huge calculation cost and makes the computation cannot be done parallelly. If new computation methods can solve these two problems while provide similar results as the current PMI network calculation procedure, it will be great help for correlation analysis of biological data.

## Conclusions

Our findings confirm that PMI is a precise and robust correlation measure for transcriptome data. The theoretical advantage of PMI direct association over PCC indirect association does stand for a higher biological importance of the edges in the two types of correlation networks. We believe this advantage is applicable to other types of biological data. PMI can be easily used to refine results from the level of modules to obtain further interpretation at the level of variable pairs. An optimal PMI network is useful in highlighting the essential parts of the correlation in one module. Although the calculation of the optimal PMI network for a genome-scale data is incomplete, our results suggest it is worth of using PMI to focus on parts with high strength correlation patterns. The advantage of PMI direct association makes it especially suitable for cancer data analysis. PMI-based correlation analysis can provide more valuable targets from the detailed correlation pattern of biological systems.

## Materials and methods

### Data selection and preprocess

We use two parts of cancer transcriptome dataset for two different purposes.

In the first part, we try to intuitively represent the characters of the PMI network. We want to find the differences between the PMI network and the PCC network and determine whether the PMI network more robustly captures the real correlation pattern. So, we need a dataset with the same set of variables but from different biological conditions. We selected 10 mRNA expression datasets of breast cancer from Gene Expression Omnibus (GEO) and ArrayExpress database (Table 2). The corresponding sample information is downloaded from ArrayExpress [13, 14]. All the 10 datasets are based on Affymetrix Hgu133plus2 platform [15–24]. The raw data from each dataset is normalized by RMA algorithm and batch-corrected by the removeBatchEffect function from R package Limma [25, 26]. Preprocessed data is separated by tumor grades and estrogen receptor (ER) status into 7 groups. Two cancer progressions are defined based on ER status in which the normal group is used twice (Table 3).

**Table 2:** Access numbers and numbers of selected samples of first part analysis

Dataset	Samples
E-MTAB-5724	515
GSE36774	107
GSE42568	118
GSE47109	208
E-TABM-276	10
GSE7904	7
GSE22544	4
GSE45827	11
GSE54002	16
GSE65194	11

**Table 3:** Numbers of samples for the two cancer progressions

Stage	ER negative	ER positive
Normal	76	
CancerStage1	46	332
CancerStage2	56	325
CancerStage3	55	117

In the second part, we use 6 TCGA cancer datasets from XenaBrowser [27]. To reduce the data heterogeneity, we split each dataset by cancer subtype. The criterion for cancer subtype definition come from the clinical information. We use the main cancer type code plus several numbers to refer to each cancer subtype; the digits of numbers equal to the attributes used to divide cancer subtypes. All cancer subtypes have zero as the first number, which means they are limited to "primary tumor" in the "sample_type" attribute of TCGA clinical information. BRCA uses 3 more attributes; they are “breast_carcinoma_estrogen_receptor_status” (0 = negative, 1 = positive), “breast_carcinoma_progesterone_receptor_status” (0 = negative, 1 = positive), and “lab_proc_her2_neu_immunohistochemistry_receptor_status” (0 = negative, 1 = mid type, 2 = positive). COAD uses 2 more attributes; the first is "CDE_ID_3226963" attribute (microsatellite instability, 0 = stable, 1 = low instable, 2 = high instable), and the second is "histological_type" attribute which limits samples to normal COAD. Mucinous COAD is filtered. ESCA uses "histological_type" attribute (0 = NOS, 1 = ESCC). LUAD uses "Expression_Subtype" attribute (0 = Bronchioid, 1 = Squamoid, 2 = Magnoid). LUSC uses "histological_type" attribute and is limited to "NOS". STAD uses "CDE_ID_3226963" attribute (microsatellite instability, 0 = stable, 1 = low instable, 2 = high instable). For example, BRCA0000 means the value of “sample_type” is “primary tumor”, the values of “breast_carcinoma_estrogen_receptor_status”, “breast_carcinoma_progesterone_receptor_status” and “lab_proc_her2_neu_immunohistochemistry_receptor_status” are all “negative”. The sample sizes of each cancer subtype and each type of data are listed in Table 4. Sample groups with more than 20 samples are used in the following analysis.

**Table 4:** The numbers of samples for cancer subtypes

CancerSubtype	GeneExpressionSamples	CNVSamples	DMSamples
BRCA0000	116	112	83
BRCA0001	32	32	24
BRCA0002	37	37	17
BRCA0010	8	8	7
BRCA0011	4	4	4
BRCA0012	3	3	3
BRCA0100	65	65	49
BRCA0101	20	19	16
BRCA0102	23	23	9
BRCA0110	369	366	253
BRCA0111	123	118	98
BRCA0112	100	98	64
COAD000	156	248	162
COAD010	41	67	41
COAD020	40	59	41
ESCA00	89	88	89
ESCA01	95	96	96
LUAD00	89	89	69
LUAD01	78	78	68
LUAD02	63	63	48
LUSC00	479	479	352
STAD00	276	294	271
STAD01	59	63	56
STAD02	79	83	68

### PMI network calculation

The mathematical definition of PMI is as follow. X and Y are two scalar variables and Z is an (n − 2)-dimensional vector (n > 2). Then, the PMI between variables X and Y given Z is defined as.

$PMI\left( {X;Y|Z} \right) = D\left( {p\left( {x,y,z} \right)||p^{*} \left( {x|z} \right)p^{*} \left( {y|z} \right)p\left( z \right)} \right),$where p(x,y,z) is the joint probability distribution of X, Y and Z, and D(p(x, y, z)||p^*^(x|z)p^*^(y|z)p(z)) represents the extended KL divergence from p(x,y,z) to p^*^(x|z)p^*^(y|z)p(z). The p^*^(x|z) and p^*^(y|z) are defined as.

$p^{*} \left( {x|z} \right) = \mathop \sum \limits_{y} p\left( {x|z,y} \right)p\left( y \right), p^{*} \left( {y|z} \right) = \mathop \sum \limits_{x} p\left( {y|z,x} \right)p\left( x \right),$and the extended KL divergence is defined as

$$D\left( {p\left( x \right),q\left( x \right)} \right) = \mathop \sum \limits_{x} p\left( x \right)\log \frac{p\left( x \right)}{{q\left( x \right)}}.$$

**Fig. 7:**
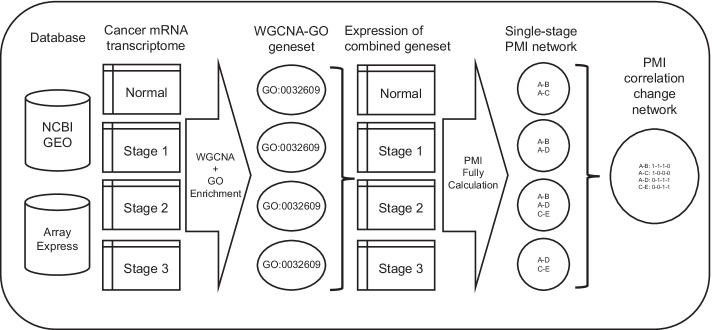
The generation of a PMI correlation change network. The cancer mRNA transcriptome data from databases is split into normal group and groups of 3 cancer stages. The WGCNA and GO enrichment are ran on each group of data separately. For a GO term enriched in all 4 group along a cancer progress, the gene sets from 4 groups are different, they are combined into one gene set. The expression of the combined gene set of a GO term is used for PMI fully calculation, the resulting networks of 4 groups along a cancer progress are combined into one network, which is the PMI correlation change network

The PMI network calculation starts with a fully connected network, and gradually removes edges by a preset threshold. To obtain the optimal PMI network for a given data, the calculation scans a range of the thresholds from low to high, until the resulting networks tend to be stable [2]. When the input data is large, the calculation complexity for an optimal calculation will be too high to finish. To overcome this mathematical unsolvable problem, we make a compromise on the size of data or the completeness of optimization. In the first part, we use PMI to show the change of correlation pattern in a small gene set along the progress of cancer, the workflow is shown in Fig. 7. We use a WGCNA module extraction coupled with WebGestalt GO enrichment analysis method to separate the whole genome into small gene sets [1, 4]. These small gene sets are internally correlated with GO-based functional annotation. We select some of them and calculate the optimal PMI networks. Then, by combining single stage networks along one cancer progression, we got the PMI correlation change network for the corresponding GO term. In a correlation change network, one edge is a combination of 1–4 single-stage edges from the 4 stages of one cancer process, the stage number of 4 stages are defined as 1–4. The stage combination of an edge is marked as four 0/1 connected by hyphens, which is referred as the existence pattern. For nodes in the correlation change network, the mean stage value is defined as the average value of all the stage numbers of the single-stage edges of one node. In the second part, we focus on the connection between network edges and omics defined gene-pair relationships. The workflow is shown in Additional file 1: Fig. S2. Separating the whole genome into gene sets will lead to some genes missed, but the whole genome contains too many variables to fulfill optimal PMI calculation. So, we use the whole genome data as input, starting with a high threshold, and try to get as many as edges in the limited time for each cancer subtype.

### Comparable PCC network

To understand the difference between PMI and PCC in the context of analysis of biological data, we need to construct a series of PCC networks based on each PMI network. The PCC networks use similar standards to the corresponding PMI network, including using the strength of correlation to select the top several correlations among a set of genes. For each PMI network, we construct 3 types of comparable PCC networks. Type 1 PCC network selects edges from all gene pairs within the PMI gene set by descending absolute PCC value until it has the same number of edges with the corresponding PMI network. Type 2 PCC network uses the same method until it covers all the genes in the PMI network. Type 3 PCC network considers each PMI edge separately. For one PMI edge Gene1-Gene2 from one PMI network, if another gene, Gene3, from the PMI network has higher absolute PCC values with both Gene1 and Gene2 than the absolute PCC value of Gene1 with Gene2, then edge Gene3-Gene1 and edge Gene3-Gene2 are both included into the edges of comparable PCC network. Edges in Type 3 PCC network may not exist in the PMI network; these edges are called PCC-only edges. PCC-only edges have higher PCC values than those of the reference PMI edges when they are selected. However, they are removed in the PMI network. The biological characters of PCC-only edges may be the key for explaining the difference between PMI correlation and PCC correlation in the gene expression pattern along with the cancer progression. Type 1 and type 2 PCC networks are only used to compare the network sparsity between PMI and PCC. Type 3 PCC networks are used to compare the difference between PMI and PCC connecting to omics defined relationships.

### Omics defined gene-pair relationships

We need to compare the edges from gene correlation networks with the relationships between a pair of genes defined by biological data or knowledge. We use transcriptional regulation (TR) data from the RegNetwork database [28]. Besides the transcriptional factor (TF) targeting one gene relationship, we check each gene-pair for common upper regulators (TF or microRNA) and transform the original TR data into CoTR data. TF and microRNA are checked separately. The CoTR relationship of one gene-pair is weighted by the number of common upper regulators. The protein–protein interaction (PPI) data is from the High-quality protein interactomes (HINT) database, only literature curated data is used [29]. The human genome coordinate data is downloaded from UCSC genome browser. GRCh37 version of NCBI RefSeq data is used. We calculate the distance between two transcription starting points of a pair of genes (if they are on the same chromosome) for the omics comparison (Same Chromosome Gene Distance, SCGD) [30]. The cancer dataset from XenaBrowser contains copy number variation (CNV) and DNA methylation (DM) data for each sample [27]. In consideration of the status of two genes, we convert them into CoCNV and CoDM data for the omics comparison. The CoCNV status of a pair of genes in one sample can be one of the following 5 types: gain–gain (GG), loss–loss (LL), gain–loss (GL), loss–gain (LG), or one or two of the genes are not mutated (NC). We define the DM data of one gene in one sample as the 2 numbers of low or high (L or H) methylation CpG islands. The CoDM status of a gene-pair in one sample is described by 4 values (LL, HH, LH, HL). Which are the products of 2 values of 2 genes cross timed. The CoCNV and CoDM of one gene-pairs are weighted and normalized by the number of samples with the corresponding mutation.

### Comparison of PMI with PCC

We assume that the correlation pattern of gene expression stems from the omics defined gene-pair relationships. We therefore match gene-pairs from PMI/PCC networks in previous steps with each type of gene-pair relationships and compare the recalling statistics between PMI network and comparable PCC network. We use the word “recall” to refer the gene-pair of a PMI edge is also a record in each omics data. For example, Gene1 and Gene2 have PPI interaction record from database, a PMI network contains the Gene1-Gene2 edge, then the PMI network recalls the Gene1-Gene2 PPI; Gene3 and Gene4 have one or more samples with both gene amplification mutation, a PMI network contains the Gene3-Gene4 edge, then the PMI network recalls the Gene3-Gene4 CoCNV-GG. Each network in each omics has a Z-score about the number of recalled gene-pairs. The null distribution was calculated by randomly select the same number of gene-pairs from all gene-pairs and check the numbers of recall for 100 times. For CoTR, CoCNV and CoDM, their records can be weighted by one or more record attributes, so they have more than one types of Z-scores. A CoTR record Gene1-Gene2 is weighted by the number of regulators (TF or miRNA) targeting both Gene1 and Gene2. A CoCNV or CoDM record is weighted by the number of samples with the type of CoCNV or CoDM. Because a CNV mutation record can be ± 1 (low level loss or amplification) or ± 2 (high level loss or amplification), so a CoCNV record can be further weighted by the absolute value of the product of the two mutation levels of two genes.

### Cancer survival analysis

We collect gene-pairs with CoCNV or CoDM relationships from the PMI networks and use them to divide patients. We use the disease specific survival (DSS) data and perform univariate survival analysis with Python lifelines package [31]. The survival curves are drawn by Python matplotlib package [32]. For one gene-pair, the CoCNV data divide the patients into 5 groups: GG, LL, GL, LG and NC. Every 2 groups with more than 20 samples are compared. A CoCNV survival factor is marked, for example as “Gene1-Gene2 GG > LL”, while the CoDM data of one gene-pair consists of 4 values: HH, LL, HL, LH. CoDM values with normalized mean average deviation higher than 0.25 are chosen. We use the median of one chosen CoDM value as threshold to divide patients into two groups, then perform the survival analysis. So, a CoDM survival factor is marked as “Gene1-Gene2 HH-low > HH-high”. Survival factors with log rank test p-value lower than 0.05 are considered as significant. We also perform similar survival analysis with single gene CNV/DM or the expression of one or two genes and compare their efficiency with the gene-pair-based factors. Then, we build a simple scoring system to classify patients with gene-pairs in significant CoCNV/CoDM factors. For a given set of CoCNV/CoDM survival factors, the survival score of a patient is calculated by summing up the influence of all gene-pairs,

$${\textit{Score}} = \sum \limits_{GP} I_{gp} ,$$

where I_gp_ is depended on the relative survival status in the gp survival factor. For CoCNV survival factors, if one type is NC, only the other type is considered has impact on survival. For example, a gene-pair Gene1-Gene2 has only one CoCNV survival factor GG > NC,

$$I_{gp} = \left\{ {\begin{array}{*{20}c} {1 \left( {{\text{If Gene}}1 - {\text{Gene}}2 {\text{in patient is GG}}} \right)} \\ {0 \left( {{\text{If Gene}}1 - {\text{Gene}}2 {\text{in patient is NC}}} \right)} \\ \end{array} } \right..$$

If another gene-pair Gene3-Gene4 has CoCNV survival factors LL > NC, NC > GG, LL > GG and GL > GG,

$$I_{gp} = \left\{ {\begin{array}{*{20}l} {1 \left( {{\text{If patient Gene}}3 - {\text{Gene}}4 {\text{in is LL or GL}}} \right)} \hfill \\ {0 \left( {{\text{If Gene}}3 - {\text{Gene}}4 {\text{in patient is NC}}} \right)} \hfill \\ { - 1 \left( {{\text{If Gene}}3 - {\text{Gene}}4 {\text{in patient is GG}}} \right)} \hfill \\ \end{array} } \right..$$

For CoDM survival factor, each CoDM type is analyzed separately. For example, a gene-pair has CoDM HH-high > HH-low survival factor,

$$I_{gp} = \left\{ {\begin{array}{*{20}c} {1 \left( {{\text{If patient is HH}} - {\text{high}}} \right)} \\ { - 1 \left( {{\text{If patient is HH}} - {\text{low}}} \right)} \\ \end{array} } \right..$$

Patients with higher score have better survival. Patients are divided into 3 groups by the CoCNV/CoDM scores then perform survival analysis. To assess if the distribution of the gene-pairs of a set of survival factors on the PMI network has influence on the survival classification, we choose 3 sets survival factors for CoCNV or CoDM, the first set has survival factors from isolated edges, the survival factors in the second set construct small network modules, the third set is the first two sets combined.

To confirm that the CoCNV/CoDM survival scores provide additional information about the survival classification as compared to other clinical covariates, we performed Cox multivariate analysis with the CoCNV/CoDM survival scores and clinical covariates. For the 3 cancer subtypes we used for survival analysis, clinical covariates gender, age_at_initial_pathologic_diagnosis, pathologic_M, pathologic_N, pathologic_T and pathologic_stage are chosen. The raw values of pathologic T/N/M/stage are simplified and converted to integers 0/1/2/3/4. Gender is used as covariate only for LUSC, and BRCA only use samples from female patients. We perform 100 times of 80% subsample, remodel on two models of LUSC00 and calculate the mean and standard deviation of the *p*-values of each covariate to confirm the consistency of results.

### Network visualization

All networks are visualized by Cytoscape with default preferred layout and manual adjustment [33].

## Supplementary Information

**Additional file 1.** **Fig S1 and Fig S2.** Fig S1 is the networks of CoDM PMI and PCC survival factors of three cancer subtypes. Fig S2 is the flow chart of omics comparison and survival analysis.

**Additional file 2.** **Supplementary Table S1.** PMI/PCC networks recall TR relationships.

**Additional file 3.** **Supplementary Table S2.** PMI/PCC networks recall TFCoTR relationships.

**Additional file 4.** **Supplementary Table S3.** PMI/PCC networks recall miRCoTR relationships.

**Additional file 5.** **Supplementary Table S4.** PMI/PCC networks recall SCGD relationships.

**Additional file 6.** **Supplementary Table S5.** PMI/PCC networks recall PPI relationships.

**Additional file 7.** **Supplementary Table S6.** Zscores of PMI networks recall CoCNV relationship.

**Additional file 8.** **Supplementary Table S7.** Zscores of PCC networks recall CoCNV relationships.

**Additional file 9.** **Supplementary Table S8.** PMI/PCC networks recall CoCNV GG relationships.

**Additional file 10.** **Supplementary Table S9.** PMI/PCC networks recall CoCNV LL relationships.

**Additional file 11.** **Supplementary Table S10.** Zscores of PMI/PCC networks recall CoDM relationships.

**Additional file 12.** **Supplementary Table S11.** PMI/PCC networks recall CoDM HH relationships.

**Additional file 13.** **Supplementary Table S12.** PMI/PCC networks recall CoDM HL relationships.

**Additional file 14.** **Supplementary Table S13.** PMI/PCC networks recall CoDM LH relationships.

**Additional file 15.** **Supplementary Table S14.**PMI/PCC networks recall CoDM LL relationships.

**Additional file 16.** **Supplementary Table S15.** PMI recalled CoCNV survival analysis.

**Additional file 17.** **Supplementary Table S16.** PMI recalled CoDM survival analysis.

**Additional file 18.** **Supplementary Table S17.** Cox multivariate analysis for BRCA0000 and BRCA0112.

**Additional file 19.** **Supplementary Table S18.** Cox multivariate analysis for LUSC00.

**Additional file 20** **Supplementary Table S19.** The p-value distribution of randomly subsampled LUSC00 Cox multivariate models using only clinical covariates.

**Additional file 21** **Supplementary Table S20.** The p-value distribution of randomly subsampled LUSC00 Cox multivariate modules using clinical covariates and both CoCNV and CoDM scores.

**Additional file 22** **Supplementary Table S21.** Comparison of survival classification using single gene CNV and two gene CoCNV.

## Data Availability

The datasets analyzed in the current study are available in the Gene Expression Omnibus database https://www.ncbi.nlm.nih.gov/geo/, accession numbers GSE7904, GSE22544, GSE36774, GSE42568, GSE45827, GSE47109, GSE54002 and GSE65194, and ArrayExpress database https://www.ebi.ac.uk/arrayexpress/, accession numbers E-MTAB-5724 and E-TABM-276, and UCSC XenaBrowser https://xenabrowser.net/datapages/ for the TCGA cancer datasets of BRCA, COAD, ESCA, LUAD, LUSC and STAD.

## Abbreviations

PMI: Part mutual information
PCC: Pearson correlation coefficient
WGCNA: Weight correlation network analysis
GO: Gene ontology
TR: Transcriptional regulation
CoTR: Co-transcriptional regulation
PPI: Protein–protein interaction
SCGD: Same chromosome gene distance
CNV: Copy number variation
DM: DNA methylation
