# Supplementary material for: Gene co-expression network based on part mutual information for gene-to-gene relationship and gene-cancer correlation analysis
Source: BMC Bioinformatics. 2022 May 24;23:194. doi: 10.1186/s12859-022-04732-9 (PMC9128248; doi:10.1186/s12859-022-04732-9)
Supplement: Supplementary file 1 — Additional file 1. Fig S1 and Fig S2. Fig S1 is the networks of CoDM PMI and PCC survival factors of three cancer subtypes. Fig S2 is the flow chart of omics comparison and survival analysis. [file 12859_2022_4732_MOESM1_ESM.pptx]

## Slide 1
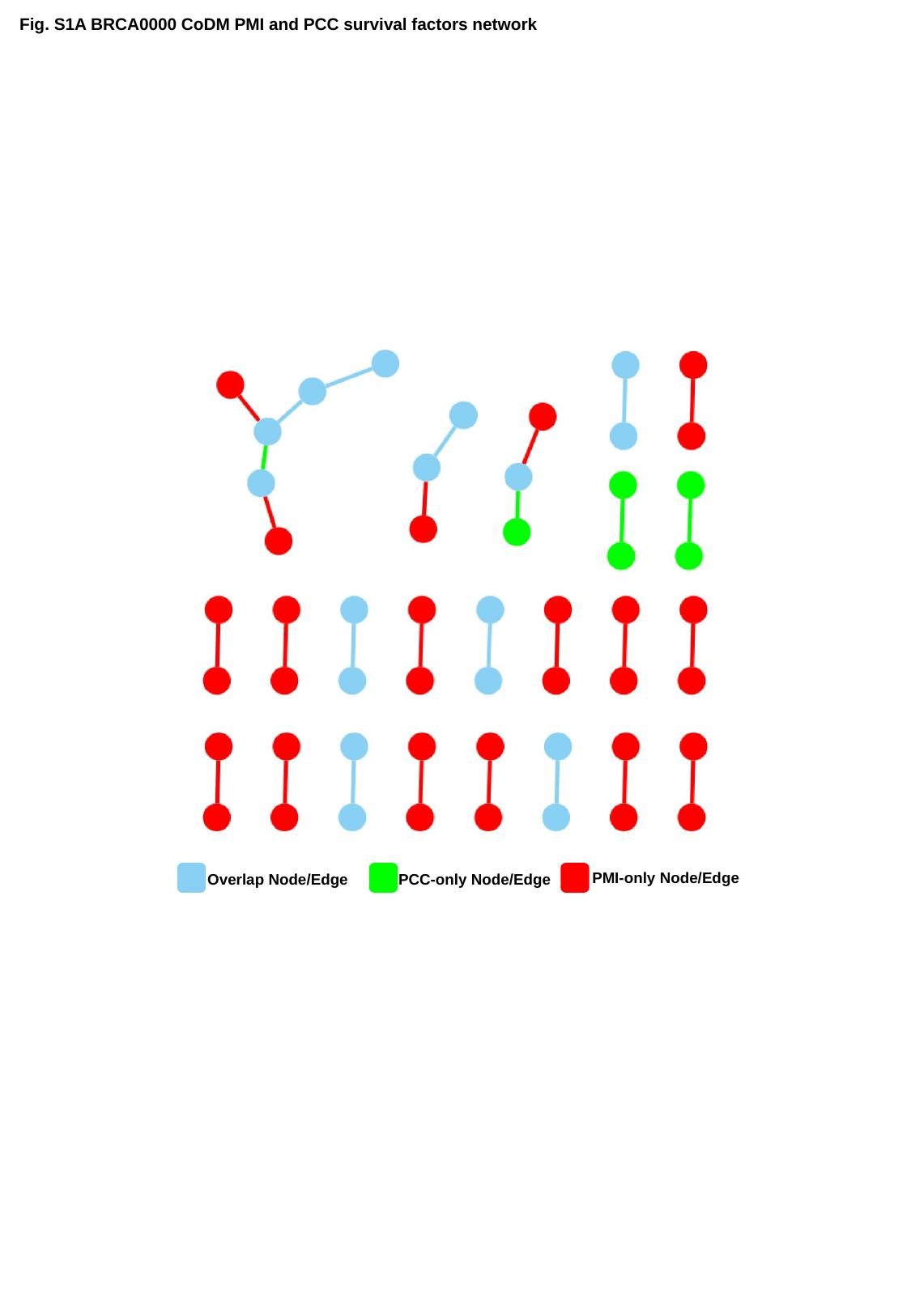

Fig. S1A BRCA0000 CoDM PMI and PCC survival factors network
PMI-only Node/Edge
Overlap Node/Edge
PCC-only Node/Edge

## Slide 2
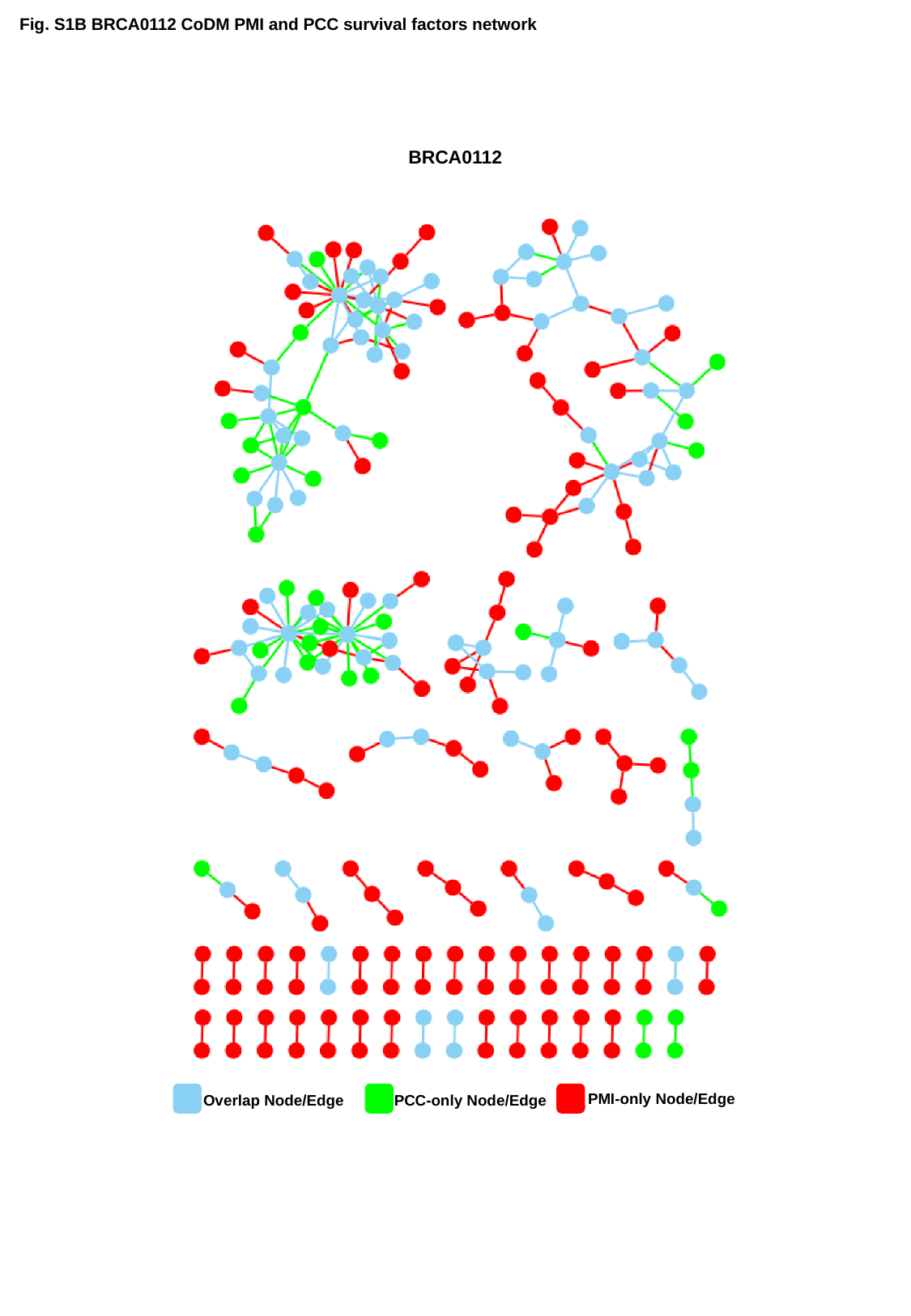

Fig. S1B BRCA0112 CoDM PMI and PCC survival factors network
BRCA0112
PMI-only Node/Edge
Overlap Node/Edge
PCC-only Node/Edge

## Slide 3
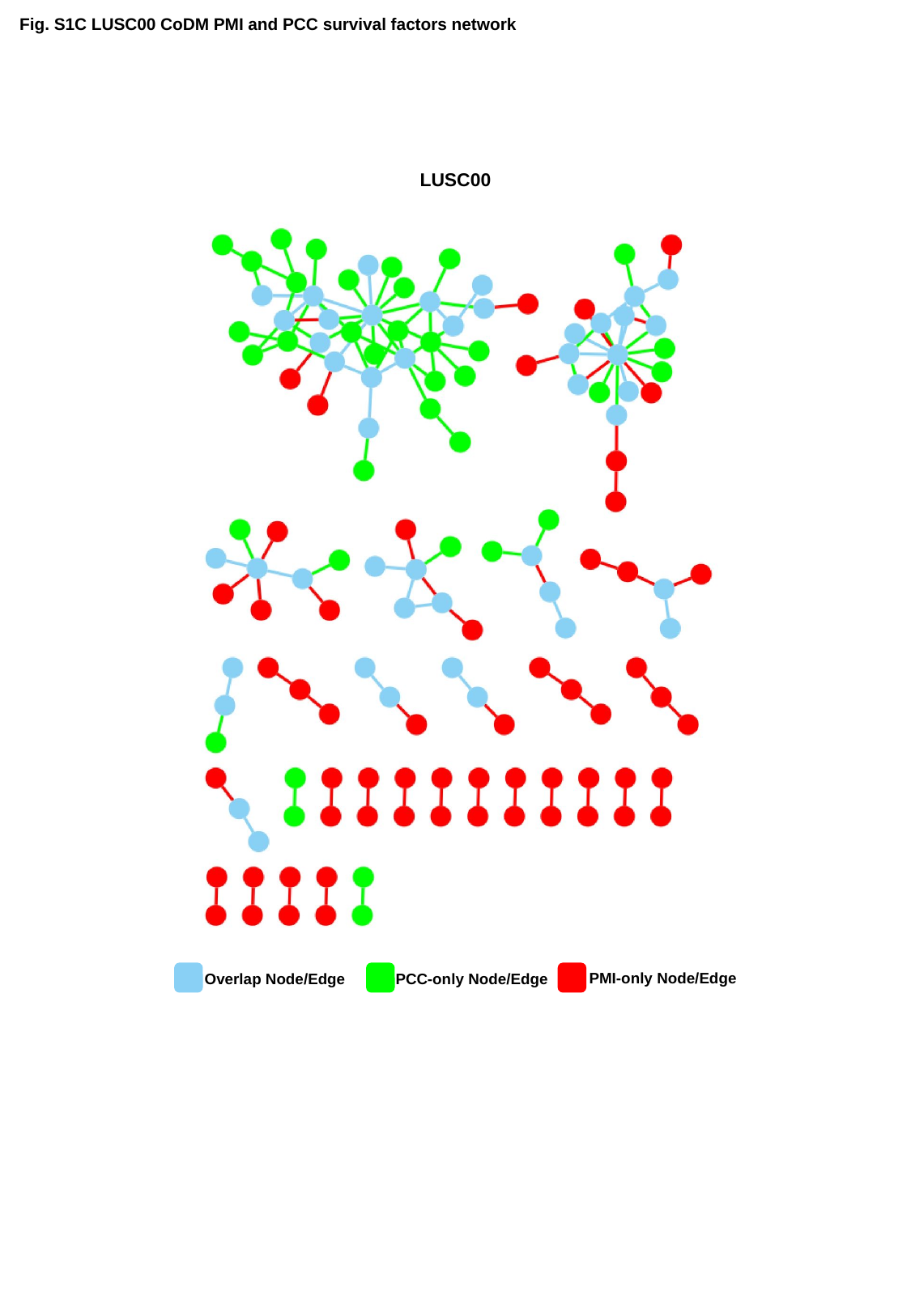

Fig. S1C LUSC00 CoDM PMI and PCC survival factors network
LUSC00
PMI-only Node/Edge
Overlap Node/Edge
PCC-only Node/Edge

## Slide 4
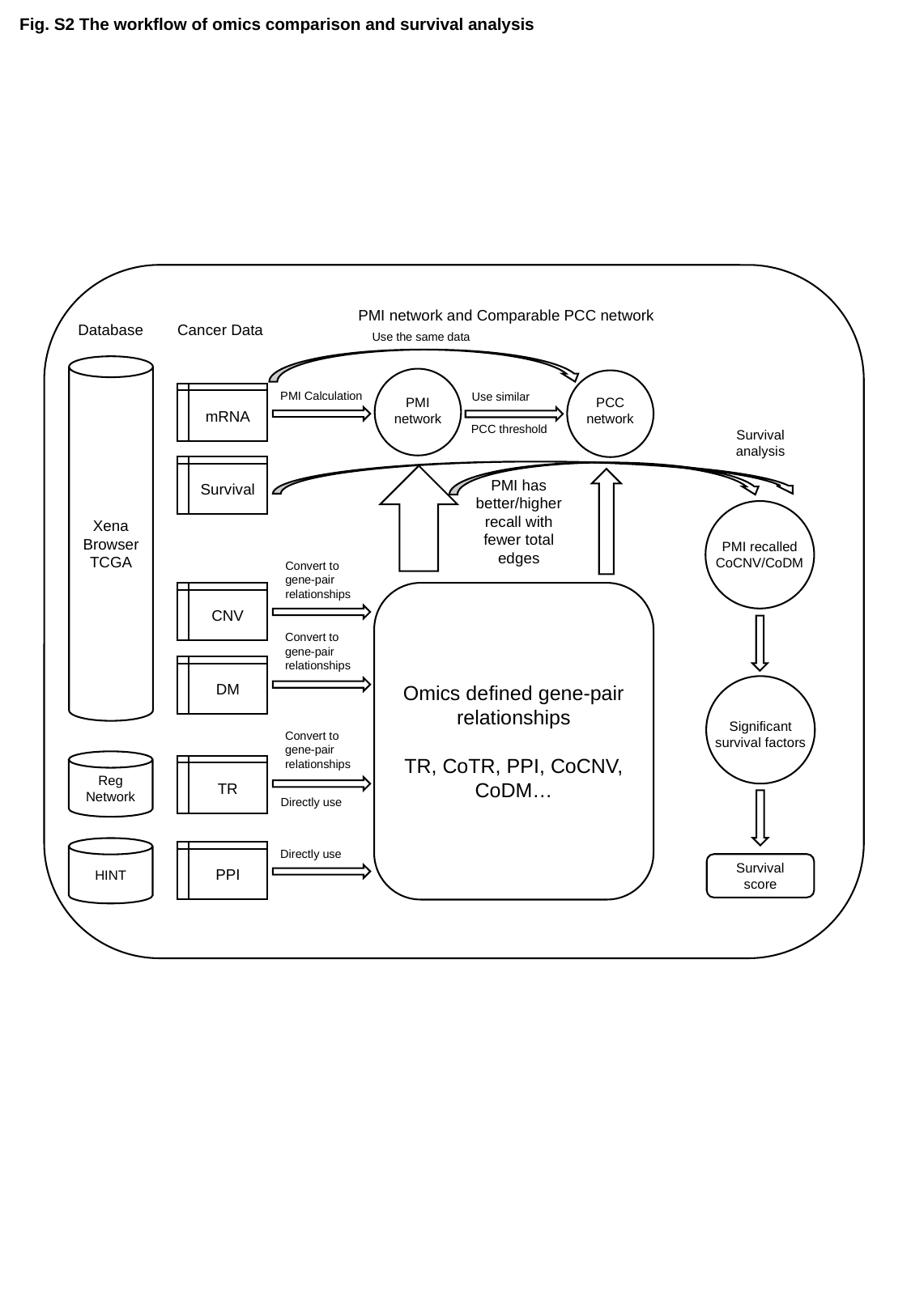

Fig. S2 The workflow of omics comparison and survival analysis
PMI network and Comparable PCC network
Cancer Data
Database
Use the same data
Xena
Browser
TCGA
PMI Calculation
Use similar
mRNA
PMI network
PCC network
PCC threshold
Survival analysis
Survival
PMI has better/higher recall with fewer total edges
PMI recalled
CoCNV/CoDM
Convert to gene-pair
relationships
CNV
Omics defined gene-pair relationships
TR, CoTR, PPI, CoCNV, CoDM…
Convert to gene-pair
relationships
DM
Significant survival factors
Convert to gene-pair
relationships
Reg
Network
TR
Directly use
HINT
Directly use
PPI
Survival score
